# Kinetic Characterization and Allosteric Inhibition of the *Yersinia pestis* 1-Deoxy-D-Xylulose 5-Phosphate Reductoisomerase (MEP Synthase)

**DOI:** 10.1371/journal.pone.0106243

**Published:** 2014-08-29

**Authors:** Amanda Haymond, Chinchu Johny, Tyrone Dowdy, Brandon Schweibenz, Karen Villarroel, Richard Young, Clark J. Mantooth, Trishal Patel, Jessica Bases, Geraldine San Jose, Emily R. Jackson, Cynthia S. Dowd, Robin D. Couch

**Affiliations:** 1 Department of Chemistry and Biochemistry, George Mason University, Manassas, Virginia, United States of America; 2 Department of Chemistry, George Washington University, Washington DC, United States of America; George Mason University, United States of America

## Abstract

The methylerythritol phosphate (MEP) pathway found in many bacteria governs the synthesis of isoprenoids, which are crucial lipid precursors for vital cell components such as ubiquinone. Because mammals synthesize isoprenoids via an alternate pathway, the bacterial MEP pathway is an attractive target for novel antibiotic development, necessitated by emerging antibiotic resistance as well as biodefense concerns. The first committed step in the MEP pathway is the reduction and isomerization of 1-deoxy-D-xylulose-5-phosphate (DXP) to methylerythritol phosphate (MEP), catalyzed by MEP synthase. To facilitate drug development, we cloned, expressed, purified, and characterized MEP synthase from *Yersinia pestis*. Enzyme assays indicate apparent kinetic constants of K_M_^DXP^ = 252 µM and K_M_^NADPH^ = 13 µM, IC_50_ values for fosmidomycin and FR900098 of 710 nM and 231 nM respectively, and K_i_ values for fosmidomycin and FR900098 of 251 nM and 101 nM respectively. To ascertain if the *Y. pestis* MEP synthase was amenable to a high-throughput screening campaign, the Z-factor was determined (0.9) then the purified enzyme was screened against a pilot scale library containing rationally designed fosmidomycin analogs and natural product extracts. Several hit molecules were obtained, most notably a natural product allosteric affector of MEP synthase and a rationally designed bisubstrate derivative of FR900098 (able to associate with both the NADPH and DXP binding sites in MEP synthase). It is particularly noteworthy that allosteric regulation of MEP synthase has not been described previously. Thus, our discovery implicates an alternative site (and new chemical space) for rational drug development.

## Introduction

Referred to as “The Great Mortality” by contemporaries, Black Death irrevocably changed the social and economic structure of 14^th^ century Europe, killing one-third of the Western European population [1]. Black Death, an outbreak of the plague, was caused by the Gram negative bacterium *Yersinia pestis* [1], [2]. In light of its high morbidity/mortality rate, ease of dissemination, associated emergency response procedures, and significant social impact, *Y. pestis* is now categorized by the US Centers for Disease Control and Prevention (CDC) as a Category A biological threat agent (i.e. an agent of greatest concern). Our vulnerability to outbreaks of infectious disease is further underscored by the 2009 H1N1 swine flu pandemic, the 2003 SARS outbreak, the 2001 anthrax letter attacks, and the 1984 Rajneeshee Salmonella attacks, stressing the necessity of effective vaccines and antimicrobial/antiviral therapeutics. The ease by which antibiotic resistance can be deliberately engineered into bacteria, and the increasing prevalence of antibiotic resistant strains, also emphasizes the need for continued development of novel antibiotics against new bacterial targets.

**Figure 1:**
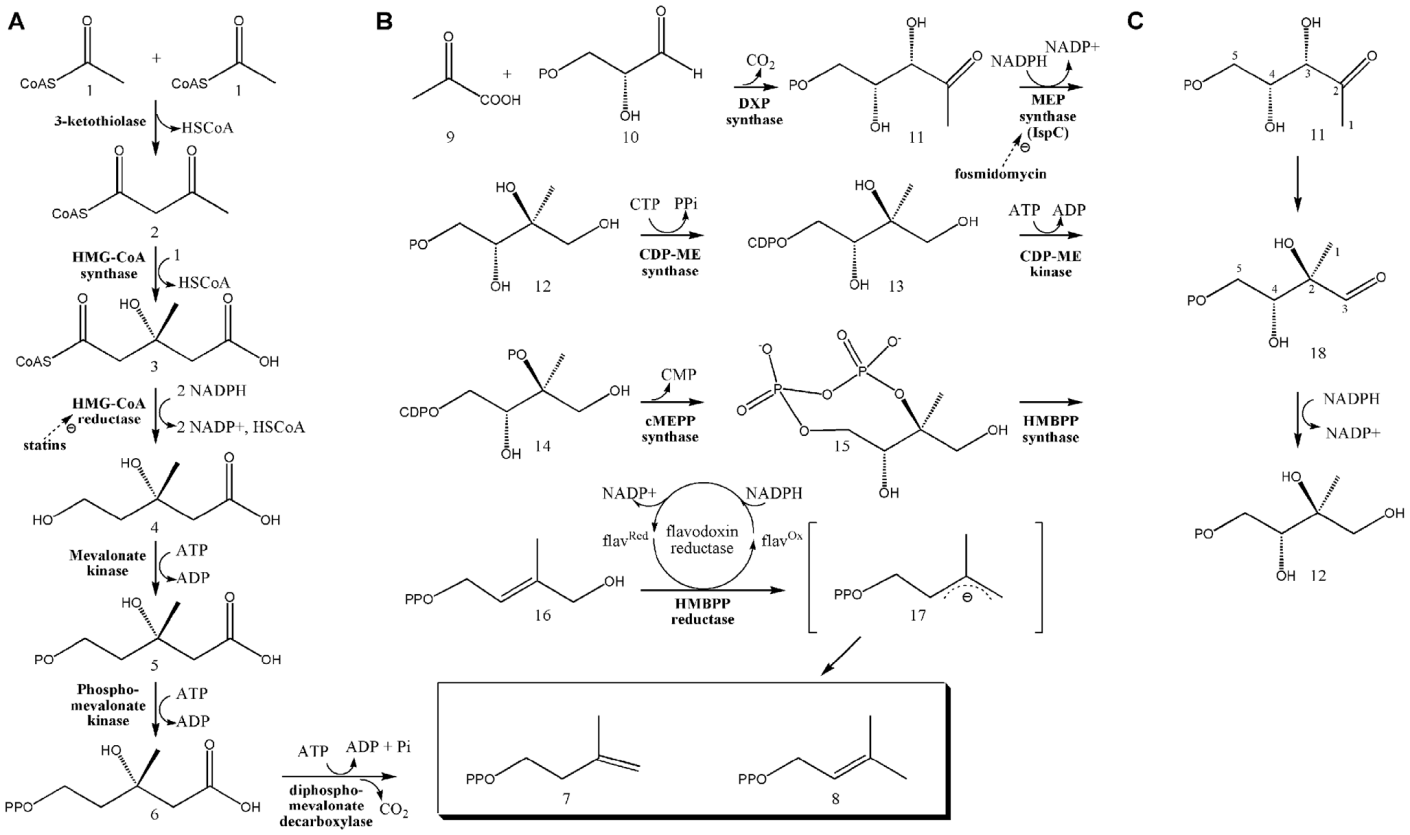
The MVA and MEP biosynthetic pathways. A) The MVA pathway is utilized by humans and other eukaryotes, archaebacteria, and certain eubacteria to produce IPP and DMAPP, the building blocks of isoprenoids. The pathway is initiated by the enzymatic condensation of 3 molecules of acetyl-CoA (1) to form 3-hydroxy-3-methylglutaryl CoA (HMG-CoA) (3), which is then reduced to MVA by HMG-CoA reductase (4) [54] [55] Subsequent phosphorylation and decarboxylation yield IPP (7) [56] [57] [58] which is converted to DMAPP (8) by an isomerase [59]. B) The MEP pathway is used by higher plants, the plastids of algae, apicomplexan protozoa, and many eubacteria, including numerous human pathogens. Pyruvate (9) is condensed with glyceraldehyde 3-phosphate (10) to yield 1-deoxy-D-xylulose 5-phosphate (DXP; (11)) [60], a branch point intermediate with a role in *E. coli* vitamin B1 and B6 biosynthesis [61] [62] [63] [64] as well as isoprene biosynthesis. In the first committed step of the *E. coli* MEP pathway, 1-Deoxy-D-xylulose 5-phosphate reductoisomerase (also called MEP synthase, Dxr or IspC) catalyzes the reduction and rearrangement of 11 to yield MEP (12) [28]. CDP-ME synthase then converts MEP into 4-(cytidine 5′-diphospho)-2-C-methyl-D-erythritol (CDP-ME; (13)). CDP-ME kinase phosphorylates CDP-ME, which is subsequently cyclized (coupled with the loss of CMP) by cMEPP synthase to yield 2-C-methyl-D-erythritol 2,4-cyclodiphosphate (15) [65] [66] [67] [68] [69]. A reductive ring opening of 15 produces 1-hydroxy-2-methyl-2-butenyl diphosphate (HMBPP; (16)) [70] [71] [72] [73] [74], which is then reduced to both IPP and DMAPP in a ∼5:1 ratio [8] [75] [76] [77] [78] [79] [80]. C) The reaction catalyzed by MEP synthase. The intermediate 2-C-methyl-D-erythrose 4-phosphate (18), produced by isomerization via cleavage of the bond between C3 and C4 and formation of a new bond between C2 and C4 [81] [82], is subsequently reduced to yield MEP (12).

Isoprenoids are a crucial family of molecules that includes compounds such as quinones and cholesterol and are involved in a number of cellular processes, from electron transport to signal transduction to the regulation of membrane fluidity. Each member of this diverse family of molecules is derived from two common building blocks; isopentenyl pyrophosphate (IPP) and its isomer dimethylallyl pyrophosphate (DMAPP), synthesized via the mevalonic acid (MVA) or methyl erythritol phosphate (MEP) pathways (Figure 1). Because the MEP pathway is exclusively utilized by many human pathogens, and knockout of MEP pathway genes has proven lethal in bacteria such as *Mycobacterium tuberculosis* [3], *Francisella tularensis* [4], *Escherichia coli* [5], and *Vibrio cholerae* [6], the MEP pathway enzymes have received considerable attention as promising targets for the development of novel antibiotics (reviewed in [7] [8] [9]).

The drug discovery process typically involves five distinguishable phases; target identification, target validation, lead molecule identification, lead optimization, and preclinical and clinical trials. The identification of lead molecules often involves the screening of a molecular library. In general, molecular libraries typically contain synthetic compounds, either rational or random in design, and/or natural products extracted from a wide variety of plant, bacteria, or fungal sources. While natural products underwent a period of reduced attention (a consequence of several factors including the substantial effort required to isolate the active component from a complex mixture, the effort required to elucidate the chemical structure of the active component, significant advances in protein structure determination by crystallography and NMR, improvements to *in silico* rational drug design using the protein structure, and the combinatorial chemistry approach to rapidly populating synthetic chemical libraries), natural product libraries are coming back in vogue, as the number of new chemical entities entering into clinical trials continues to decline. Nearly one-third of the pharmaceuticals worldwide are natural products or their derivatives. In fact, most antibacterial drugs originate from natural products, including the β-lactams, tetracyclines, aminoglycosides, chloramphenicol, cephalosporins, macrolides, lincosamides, rifamycins, streptogramins, the glycopeptides, and the lipopeptides. The blockbuster anticholesterol drug Mevacor (lovastatin), a natural product produced by the fungus *Aspergillus terreus*, inhibits HMG-CoA reductase, thereby blocking the rate limiting enzyme in the MVA isoprene biosynthetic pathway (Fig. 1) [10] [11].

Several groups have demonstrated an interest in developing small molecule inhibitors of the MEP pathway enzymes, including those that target DXP synthase [12], [13], MEP synthase (reviewed in [14]), CDP-ME synthase [15], [16], CDP-ME kinase [17], [18], cMEPP synthase [19], [20], HMB-PP synthase [21], [22], and HMB-PP reductase [23], [24]. While each of these MEP pathway enzymes is a viable target for drug development [25] the focus of this report is on MEP synthase. Herein we describe the cloning, expression, and kinetic characterization of purified *Y. pestis* MEP synthase, the first committed enzyme of the MEP isoprene biosynthetic pathway. Using known MEP synthase inhibitors, we demonstrate the effectiveness of inhibiting both the purified enzyme and liquid cultures of *Y. pestis*. We also report the outcome of an in-house high-throughput screen of both a rationally designed synthetic compound library and a natural product library, resulting in the identification of several inhibitor hits, including a completely novel class of MEP synthase inhibitors that functions via an allosteric mechanism.

## Materials and Methods

### Bacterial strains and growth conditions

The following reagent was obtained through the NIH Biodefense and Emerging Infections Research Resources Repository, NIAID, NIH: *Yersinia pestis* subsp. A1122, cultured at 37°C in Tryptic Soy Broth with 0.1% cysteine (TSB). Recombinant proteins were expressed in *Escherichia coli* BL21 CodonPlus (DE3)-RIL cells from Stratagene, La Jolla, CA that were grown at 37°C in Luria-Bertani (LB) media supplemented with 100 µg/mL ampicillin and 50 µg/ml chloramphenicol. All liquid cultures were constantly shaken at 250 rpm. Agar (1.5% wt/vol) was added to prepare solid media.

### Growth inhibition assays

The half-maximal inhibitory concentration (IC_50_) for FR900098, a known MEP synthase inhibitor [7], was determined via a dose-response plot of cell growth (OD_600_) as a function of inhibitor concentration. An overnight culture of *Y. pestis* subsp. A1122 was grown in TSB and harvested by centrifugation (15 min, 2450×g, 25°C). The resulting cell pellet was washed twice with 1 mL of TSB then diluted to an OD_600_ of 0.2. Aliquots of the culture were then dispensed into 10×1 cm foam-capped test tubes containing 2 mL of fresh TSB and FR900098 added to the tubes at the indicated concentrations. Bacterial growth was monitored over 24 hrs. Each condition was evaluated in duplicate. Nonlinear regression fitting the resulting dose-response plot was achieved using GraphPad Prism version 4.00 for Windows (GraphPad software Inc, San Diego, CA) and the equation F = 1/(1+[I]/IC_50_) where F = fractional growth and [I] = inhibitor concentration. For growth inhibition assays run with rationally designed compounds, a small volume protocol was used in which a diluted overnight culture was used to inoculate 400 µL of TSB containing the appropriate concentration of inhibitor. Cultures were grown in foam-capped, 1.5 mL microcentrifuge tubes and monitored over 24 hours. For the small volume protocol, each condition was evaluated in triplicate.

### The cloning, expression, and purification of *Y. pestis* MEP synthase

The *Y. pestis* CO92 MEP synthase gene (*ispC*) was identified in the complete genome sequence using primary sequence homology with orthologs from other organisms. Sequence alignment was accomplished using Clustal Omega, with the following reference sequence numbers and protein sequence numbers obtained from the National Center for Biotechnology Information (NCBI): *Y. pestis* (NC_003143.1, YP_002346091.1), *E. coli* (U00096.2, AAC73284.1), *M. tuberculosis* (NC_000962.3, NP_217386.2), *F. tularensis* (AJ749949.2, CAG46207.1), *V. cholerae* (NC_002505.1, NP_231885.1), *B. anthracis* (AE016879.1, AAP27179.1), *M. leprae* (NC_002677.1, NP_302094.1), and *T. pallidum* (NC_000919.1, NP_219039.1) The *Y. pestis ispC* gene was synthesized (GenScript USA Inc, Piscataway, NJ) and cloned into a pET101/D-TOPO vector, facilitating the expression of a C-terminal His-tagged protein. Restriction mapping and DNA sequencing were used to confirm the construction of the plasmid (pYpIspC). The plasmid was transformed into chemically competent *E. coli* BL21 CodonPlus (DE3)-RIL cells (Stratagene, La Jolla, CA) for protein expression.

A 10 mL overnight seed culture of *E. coli* BL21 CodonPlus (DE3)-RIL+pYpIspC was added to 1 L of LB media and incubated with shaking at 37°C and 250 rpm. Once an OD_600_ of 1.8 was achieved, protein expression was induced using 0.5 mM isopropyl β-D-thiogalactopyranoside (IPTG) and the culture was allowed to incubate for an additional 18 hours. Cells were harvested via centrifugation (4650×g, 20 min) and stored at −80°C. Protein was isolated and purified from the cells via chemical lysis and affinity chromatography. Cell lysis was achieved using Lysis Buffer A (100 mM Tris pH 8, 0.032% lysozyme, 3 mL per mg cell pellet), followed by Lysis Buffer B (0.1 M CaCl_2_, 0.1 M MgCl_2_, 0.1 M NaCl, 0.020% DNase, 0.3 mL per mg cell pellet). Centrifugation (48,000×g, 20 min) yielded the clarified cell lysate that was passed through a TALON immobilized metal affinity column (Clontech Laboratories, Mountain View, CA). The column was washed with 20 column volumes of 1× equilibrium buffer (50 mM HEPES pH 7.5, 300 mM NaCl), 10 column volumes of 1× wash buffer (50 mM HEPES pH 7.5, 300 mM NaCl, 10 mM imidazole), and 15 column volumes of 2× wash buffer (100 mM HEPES pH 7.5, 600 mM NaCl, 20 mM imidazole). The protein was then eluted with 5 column volumes of 1× elution buffer (150 mM imidazole pH 7.0, 300 mM NaCl). Buffer was exchanged with 0.1 M Tris pH 7.5, 1 mM NaCl, 5 mM DTT during concentration by ultrafiltration. Protein concentration was determined using Advanced Protein Assay Reagent (Cytoskeleton, Denver CO) with γ-globulins (Sigma-Aldrich) as the standard. Purified protein was visualized via Coomassie stained SDS-PAGE. The yield of YpIspC averaged 30 mg per 1 L shake flask. *M. tuberculosis* and *F. tularensis* MEP synthase [26] were cloned, expressed, and purified essentially as described above.

### Enzyme Assays

MEP synthase activity was assayed at 37°C by spectrophotometrically monitoring the enzyme catalyzed oxidation of NADPH (Fig. 1C), as previously described [27]. All assays were performed in duplicate. To determine the apparent K_M_ for 1 deoxy-D-xylulose 5-phosphate (DXP), 120 µL assay solutions contained 100 mM Tris pH 7.8, 25 mM MgCl_2_, 150 µM NADPH, 0.89 µM MEP synthase, and variable concentrations of DXP (Echelon Biosciences, Salt Lake City, UT). The assay solution was incubated at 37°C for 10 minutes to allow NADPH to associate with the enzyme prior to the addition of DXP. To determine the apparent K_M_ for NADPH, assays were performed with fixed DXP concentration (0.4 mM) and a variable concentration of NADPH. Nonlinear regression to the Michaelis-Menton equation enabled the determination of kinetic constants. To determine cation specificity, assays were performed with 25 mM MgCl_2_, CaCl_2_, CoCl_2_, CuCl_2_, MnCl_2_, or NiCl_2_. Assays performed with isopentenyl pyrophosphate, dimethylallyl pyrophosphate, and geranyl pyrophosphate (Echelon Biosciences, Salt Lake City, Utah) included a 10 minute preincubation with the enzyme (37°C) before addition of NADPH. The half-maximal inhibition (IC_50_) by fosmidomycin and FR900098 were determined by using a plot of enzyme fractional activity as a function of inhibitor concentration. As they are slow, tight binding inhibitors [28], fosmidomycin and FR900098 were pre-incubated with the enzyme for 10 minutes prior to the addition of the substrate. Molecular library screening for inhibitors also included this pre-incubation step.

### Molecular Modeling

The *Y. pestis* MEP synthase was homology-modeled using I-TASSER (http://zhanglab.ccmb.med.umich.edu/I-TASSER/) [29] [30] [31]. Templates were selected by I-TASSER’s threading alignment algorithm which considers predicted secondary structure features in the sequence and identifies analogous and homologous protein templates. The optimized model was then evaluated with ProQ2 (http://www.bioinfo.ifm.liu.se/ProQ2/) [32], which uses features such as atom-atom contacts, residue-residue contacts, solvent accessibility, and secondary structure information to assign an accuracy score from 0 (unreliable) to 1 (reliable). Swiss- PdbViewer 4.0 (http://spdbv.vital-it.ch/) was used to visualize and annotate the model.

### Library Screening

A rationally designed, small molecule library was compiled using compounds synthesized as described [33]. Each compound was designed from resolved crystal structures of *M. tuberculosis* MEP synthase in complex with fosmidomycin [33] [34] and contains an amide-linked or O-linked functional group.

A natural product library was compiled in house using extracts obtained from 80 different biological sources. After solvent extraction, the extracts were dried under vacuum, dispensed by mass, and resuspended in dimethyl sulfoxide (DMSO) for use in the screen. For dose-response and mechanism of inhibition plots, inhibitor concentrations are dilutions relative to the original stock solution.

## Results and Discussion

### Validating the *Y. pestis* MEP pathway as an antimicrobial target

**Figure 2:**
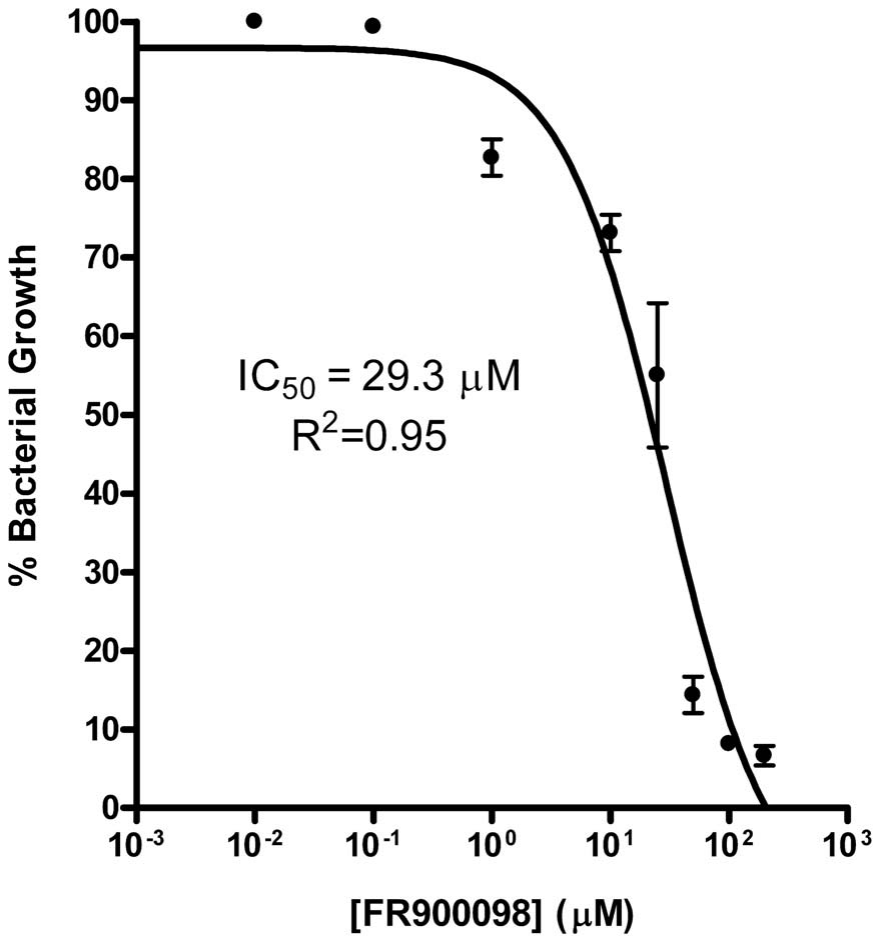
Dose-response plot of *Y. pestis* growth as a function of FR900098 concentration. Fractional growth is calculated as the ratio of cell density (OD_600_) in the presence of inhibitor to cell density in the absence of inhibitor. Nonlinear regression fitting was performed, resulting in an IC_50_ of 29 µM (6.4 µg/mL). The goodness-of-fit (R^2^) value is indicated. For comparison, the IC_50_ of ampicillin is 10.8 µM (3.8 µg/mL; see Figure S1).

The streptomycete natural products known as fosmidomycin [35] and FR900098 [36] belong to the phosphonate class of molecules and are well characterized inhibitors of MEP synthase, the enzyme responsible for catalyzing the first committed step in the MEP pathway (Fig. 1). Fosmidomycin is currently undergoing clinical trials due to its demonstrated inhibitory activity against a variety of Gram-negative and Gram-positive bacteria, as well as malaria parasites [37], [7]. The phosphonate inhibitors are actively transported into cells via a glycerol-3-phosphate transporter (GlpT, [38]), although lipophilic phosphonate prodrugs have proven highly effective MEP pathway inhibitors against pathogens lacking the GlpT protein (e.g. *Mycobacterium tuberculosis* [39] [40]). A BLAST search with the *E. coli* GlpT sequence (accession P08194) identifies a homologous transport protein in the *Y. pestis* CO92 proteome (YP_002347496; 18% identity/33% homology to the *E. coli* GlpT sequence). To evaluate the effectiveness of MEP pathway inhibition in *Y. pestis*, a growth inhibition assay was performed using FR900098. As depicted in Figure 2, FR900098 clearly inhibits *Y. pestis* proliferation in a dose-dependent manner, with half maximal inhibition (IC_50_) at 29 µM (6.4 µg/mL), comparable to the potency observed with ampicillin (Figure S1) and to the activity of fosmidomycin with *F. tularensis* (12.1 µM; [26]). Hence, MEP pathway inhibition appears to be a valid and effective means of inhibiting the propagation of *Y. pestis*. Thus, to facilitate the development of novel MEP synthase inhibitors, we next cloned and characterized the *Y. pestis* enzyme.

### Characterization of the *Y. pestis* MEP synthase

The 1197 bp MEP synthase gene (*ispC*; Gene ID: 1173888) was identified in the fully virulent *Y. pestis* subsp. *orientalis* str. CO92 genome sequence (NC_003143.1) through its homology with other known *ispC* gene sequences. The predicted amino acid sequence of the CO92 MEP synthase is identical to those predicted from the genomes of the virulent KIM 10+ and avirulent A1122 *Y. pestis* strains (NC_004088.1 and NC_017168.1, respectively), encoding a projected 43.1 kDa protein. Figure S2 displays a primary sequence alignment of the *Y. pestis* MEP synthase with several bacterial homologs, highlighting the conservation among the family of enzymes. In particular, active site catalytic residues are strictly conserved across the enzymes, as is the serine residue identified previously as a potential site of phosphoregulation [26]. Figure S3 presents a predicted tertiary structure of the *Y. pestis* MEP synthase, homology-modeled using I-TASSER. Overall, the predicted topology of the enzyme active site is highly conserved with those seen in the structurally resolved MEP synthases [41], [42]. However, subtle but notable differences are observed. Most significantly, the positioning of histidine 209, an active site residue known to coordinate the phosphonate moiety of fosmidomycin in the *M. tuberculosis* MEP synthase [43], is more akin to that observed in the *E. coli* homolog, wherein this histidine does not specifically associate with the phosphonate [44]. While the predicted structural differences may simply be a consequence of computational modeling, the slight alterations in active site topology might account for differences observed among the derived kinetic constants and the relative potency of inhibitors with the various homologs of MEP synthase (as described below).

**Figure 3:**
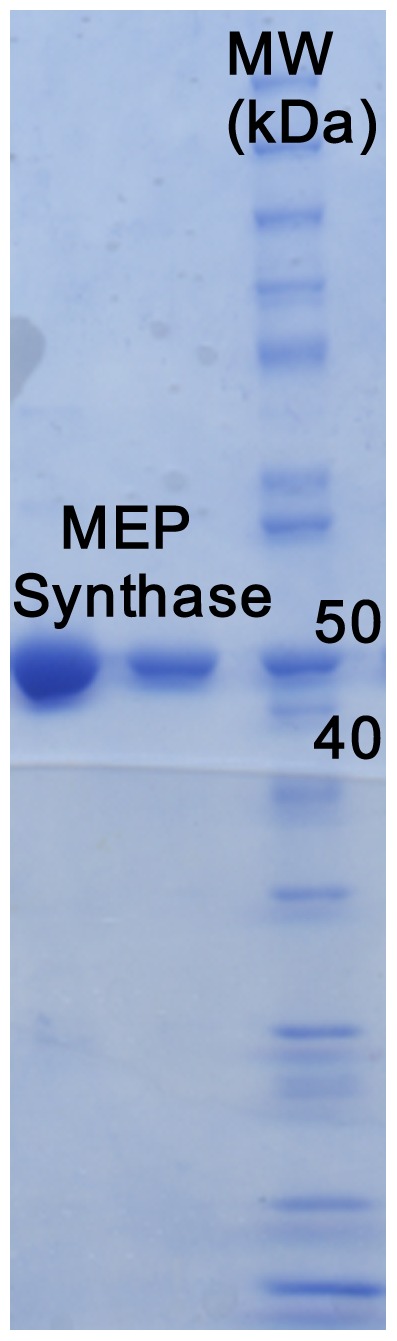
Purification of recombinant *Y. pestis* MEP synthase. A Coomassie stained SDS-PAGE shows two lanes of purified His-tagged MEP synthase alongside a molecular weight marker (MW). His-tagged MEP synthase has a predicted molecular weight of 46.7 kDa. The typical yield of purified protein averaged 30 mg per 1 L shake flask.

**Figure 4:**
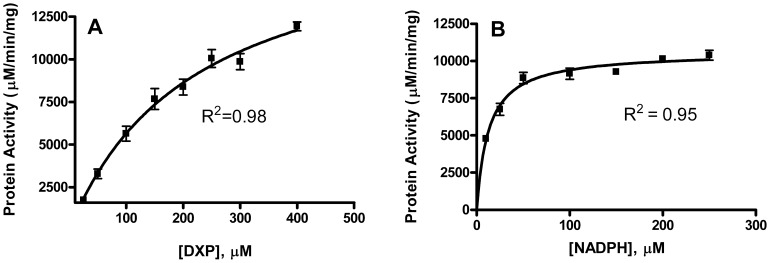
The substrate dependent catalytic activity of *Y. pestis* MEP synthase. Shown are the Michaelis-Menten plots of reaction velocity as a function of A) DXP concentration and B) NADPH concentration. Least-squares best fit of the data to the Michaelis-Menten equation produces the kinetic parameters listed in Table 1. The R^2^ value for each plot is indicated. All assays were performed in duplicate.

**Figure 5:**
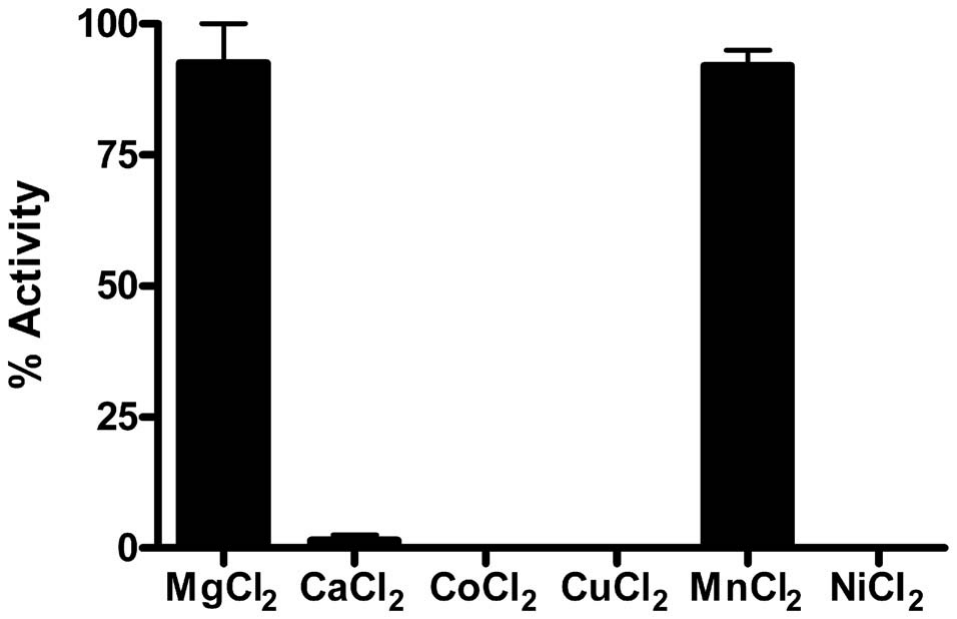
Cation specificity of *Y. pestis* MEP synthase. Enzyme assays were performed with fixed NADPH (150 µM), DXP (400 µM), and divalent cation (25 mM) concentration. *Y. pestis* MEP synthase has comparable activity with either Mg^2+^ or Mn^2+^. Assays were performed in duplicate.

To enable the enzymatic characterization of the *Y. pestis* MEP synthase, the CO92 *ispC* gene was cloned into the pET101/D expression vector, transformed into *E. coli* BL21(DE3) codon plus RIL cells, and the resulting recombinant protein was affinity purified to near homogeneity via a C-terminal histidine tag (Figure 3). The catalytic activity of the purified recombinant enzyme was determined by a spectrophotometric assay monitoring the substrate dependent oxidation of NADPH (Fig. 1C). Nonlinear regression fitting of enzyme velocity versus substrate concentration was used to determine the apparent kinetic constants (Figure 4 and Table 1). The *K_M_^app^* for 1-deoxy-D-xylulose 5-phosphate (DXP) was obtained using assays performed with a saturating concentration of NADPH (150 µM), whereas the *K_M_^app^* for NADPH was determined using assays with 400 µM DXP. In general, the recombinant *Y. pestis* MEP synthase has *K_M_^app,DXP^and K_M_^app,NADPH^* values that are comparable to those reported for homologous enzymes from other organisms (Table 1). The apparent specificity constant (*K_cat_^DXP^*/*K_M_^DXP^*) is also similar to the *Francisella*, *Mycobacterium*, and *Synechocystis* enzymes, although it is nearly 50 fold lower than that reported for the *E. coli* enzyme, predominantly due to the difference in *K_cat_^DXP^*. An evaluation of various divalent cations reveals that recombinant *Y. pestis* MEP synthase can equally use Mg^2+^ or Mn^2+^ (Figure 5), in contrast to the *F. tularensis* enzyme, which demonstrates a clear preference for Mg^2+^ [26], and the *E. coli* enzyme which can utilize Mg^2+^, Mn^2+^, or Co^2+^ [45]. The *M. tuberculosis* enzyme, initially thought to be strictly dependent upon Mg^2+^ [46], was also shown to utilize Mn^2+^ ( [47], [43]).

**Table 1:** MEP synthase Apparent Kinetic Parameters.

MEP Synthase	K_m_^DXP^ (µM)	K_m_^NADPH^ (µM)	K_cat_^DXP^ (s^−1^)	K_cat_^NADPH^ (s^−1^)	K_cat_^DXP^/K_m_^DXP^ (M^−1^ min^−1^)	IC_50_^fos^ (nM)	K_i_^fos^ (nM)	IC_50_^FR900098^ (nM)	K_i_^FR900098^ (nM)	Ref.
*Y.pestis*	221.5±34.3	12.7±1.5	1.7	1.0	4.6 x10^5^	710	968	231	170±7.10	This study
*F.tularensis*	103.7±12.1	13.3±1.5	2.0±0.09	1.3±0.04	1.2×10^6^±9×10^4^	247	98.9±4.5	230	–	[26]PM, [39]PM
*E.coli*	81–250	0.5–18	33	–	2.4×10^7^	35	21–215	35	–	[45]PM, [83]PM, [35]PM, [84]PM
*M.tuberculosis*	47	29.7	1.2	–	1.5×10^6^	80	–	160	–	[46]PM, [48]PM
*Synechocytis sp PCC6803*	170	3.5	17	–	6×10^6^	–	4	–	2	[85]PM, [86]PM

**Figure 6:**
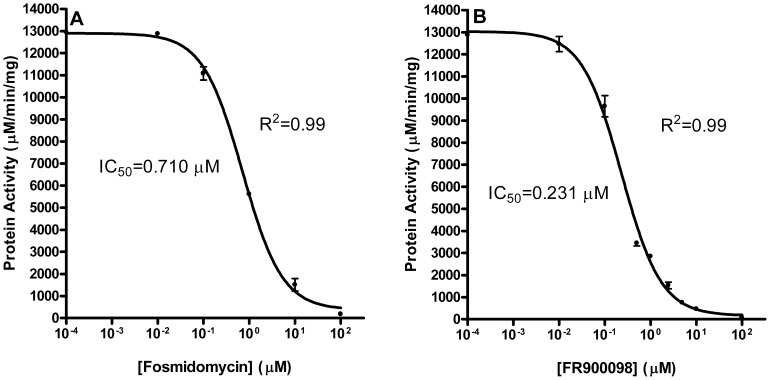
Dose-dependent inhibition of the *Y. pestis* MEP synthase. IC_50_ values were determined using A) fosmidomycin or B) FR900098. The R^2^ value for each plot is indicated. Assays were performed in duplicate.

We next determined the susceptibility of the *Y. pestis* MEP synthase to the phosphonate inhibitors fosmidomycin and FR900098. The resolved crystal structures of *M. tuberculosis* MEP synthase co-crystalized with either fosmidomycin [43] or FR900098 [34] reveal a slight steric clash among an active site tryptophan residue and the ketone methyl group of FR900098, which is reflected in the corresponding half maximal inhibitory concentration (IC_50_) of fosmidomycin (80 nM) and FR900098 (160 nM) [48]. However, this phenomenon appears unique to the *M. tuberculosis* enzyme, as FR900098 is more potent than fosmidomycin when assayed with the MEP synthase from *F. tularensis* [26], *P. falciparum* [49], and Synechocytis sp. PCC6803 [50] and is comparable in potency to fosmidomycin with the MEP synthase from *E. coli* [49] and *P. aeruginosa* [51]. In agreement with this consensus, as depicted in Figure 6, while the efficacy (E_max_) of the two inhibitors are nearly identical, FR900098 demonstrates greater potency with the *Y. pestis* MEP synthase than does fosmidomycin, with IC_50_ values of 231 nM and 710 nM, respectively. Furthermore, this preference is further apparent in comparison of the measured inhibition constants, with a *K_i_^fos^* of 968 nM and *K_i_^FR900098^* of 170 nM (Table 1 and Figure S4). Hence, while there appear to be subtle nuances in the active site topology of the *M. tuberculosis* MEP synthase that confers specificity for fosmidomycin, the *Y. pestis* MEP synthase demonstrates a clear preference for FR900098, as is also observed with the other aforementioned orthologs.

### Molecular Library Screening

We next sought to evaluate the amenability of the *Y. pestis* MEP synthase in a high throughput screening format. The quality and robustness of an enzyme assay are important considerations for the reliable screening of a molecular library, and are typically described in terms of the Z-factor [52]. Ideally, an assay should have a large dynamic range (the difference between the uninhibited and inhibited signals) and small standard deviation across replicates, which corresponds to a Z-factor score near a value of 1 (an assay with a Z-factor score between 0.5 and 1.0 is considered excellent for screening). To determine the Z-factor for the spectrophotometric assay using the *Y. pestis* MEP synthase, we fixed the DXP concentration to the K_M_, used a saturating concentration of NADPH (150 mM), and evaluated three separate lots of purified enzyme in a series of assays performed over three consecutive days. FR900098 was used as a positive control for inhibition. The Z-factor was determined to be 0.9, indicative of an assay well suited for library screening.

### Rational Library Screening

**Figure 7:**
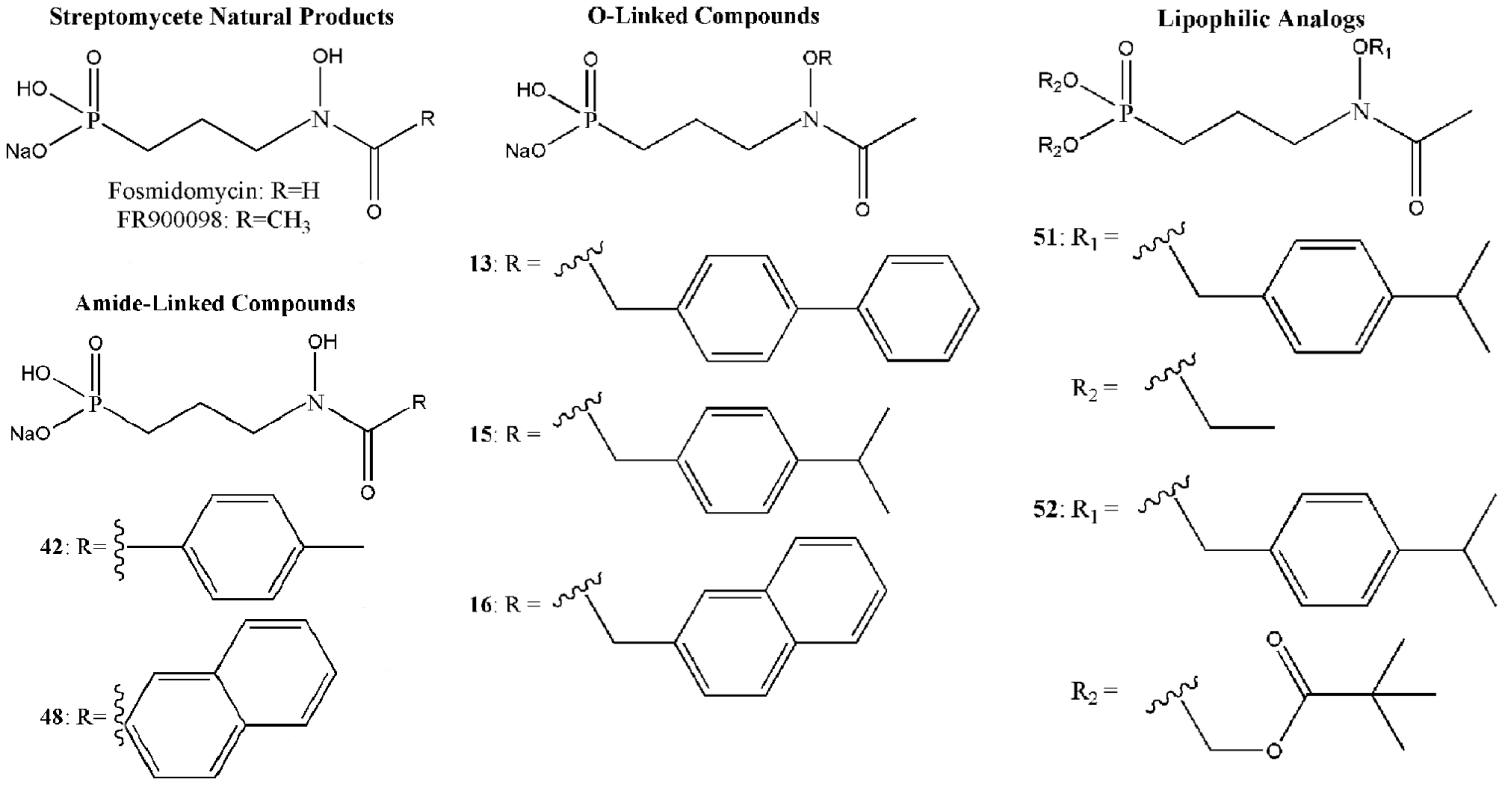
MEP synthase inhibitors. The structures of fosmidomycin, FR900098, and select rationally designed amide-linked and O-linked inhibitors are shown, including lipophilic prodrug analogs of compound **15**.

**Figure 8:**
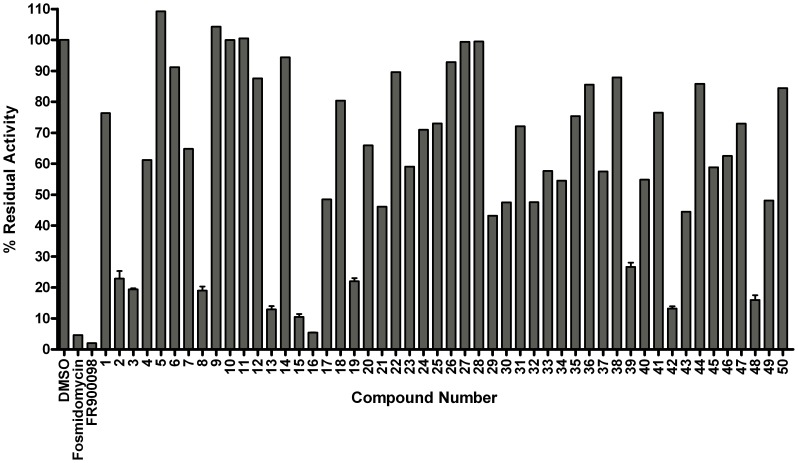
Screening a rationally designed molecular library. The *Y. pestis* MEP synthase was assayed in the presence of 100 µM of the indicated inhibitor. Residual activity is relative to the assay performed with vehicle alone (DMSO). All assays were performed in duplicate. Of those compounds tested, five inhibit the enzymatic activity by >75%, including compounds **13**, **15**, **16**, **42**, and **48**.

**Figure 9:**
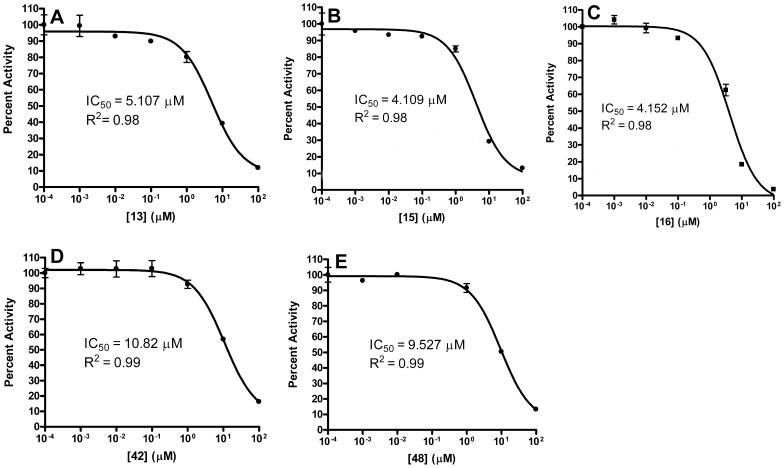
Dose-response plot of *Y. pestis* MEP synthase with the top five rationally designed inhibitors; compounds A) 13, B) 15, C) 16, D) 42 and E) 48. Assays were performed by combining the enzyme with 150 µM NADPH, followed by addition of the inhibitor. After five minute incubation at 37°C, substrate was added to initiate the reaction. The R^2^ value for each plot is indicated. The enzymatic activity is relative to an uninhibited control. All assays were performed in duplicate.

**Figure 10:**
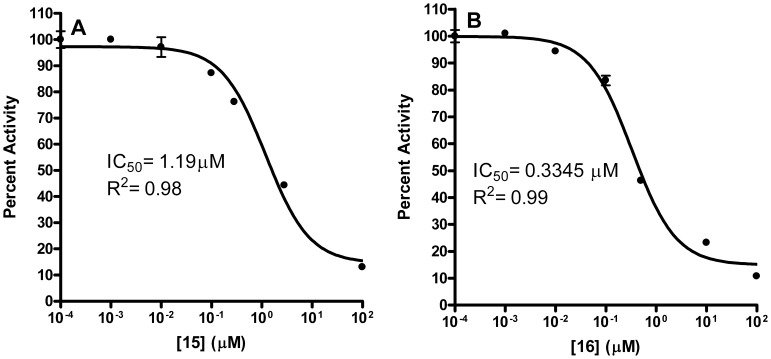
Dose-response plot of the *Y. pestis* MEP synthase when preincubated with the inhibitor. Assays were performed by combining the enzyme with either A) compound **15** or B) compound **16** and preincubating at 37°C for 10 min before addition of NADPH and DXP. All assays were performed in duplicate. Activity of the enzyme is relative to an uninhibited control.

To further assess the *Y. pestis* MEP synthase in a screening platform, we screened two pilot scale molecular libraries for inhibitory activity. The first library consists of 50 rationally designed synthetic compounds, primarily modeled on the structures of the *M. tuberculosis* MEP synthase in complex with fosmidomycin or FR900098 ( [43], [34]). As introduced elsewhere [33], the strategy for the synthesis of this library was to create novel compounds with either amide- or O-linked substituents appended to the retrohydroxamate moiety of fosmidomycin/FR900098, thereby targeting the two major binding sites in MEP synthase; the fosmidomycin/DXP site and the NADPH site, bridging these adjacent sites to yield a highly specific ligand. Select structures of the inhibitors are shown in Figure 7. As anticipated, when screening this rational library against the purified *Y. pestis* MEP synthase, several of the compounds were found to demonstrate significant inhibitory activity (>75% inhibition), as illustrated in Figure 8. The top five inhibitors were subsequently evaluated in dose-response assays (Figure 9), with compounds **15** and **16** demonstrating the greatest potency. Due to the potential for competitive bisubstrate inhibition, we also evaluated **15** and **16** by preincubating the enzyme with inhibitor prior to the addition of NADPH or DXP (in contrast to the assays depicted in Figure 9, wherein the enzyme was concomitantly exposed to NADPH and the inhibitor). As shown in Figure 10, the resulting IC_50_ values for compounds **15** and **16** improve approximately 3- and 12-fold, respectively, supportive of competitive inhibition relative to both NADPH and DXP. It is particularly noteworthy that the IC_50_ for compound **16** (0.3345 µM) approximates one half of the MEP synthase concentration used in the assay, indicative of a tight-binding inhibitor.

To further explore if compound **16** inhibits by occupying both the DXP and NADPH binding sites, we next performed inhibitor modality assays with the purified *Y. pestis* MEP synthase. Catalysis by MEP synthase involves an ordered bi bi reaction mechanism, wherein NADPH must bind to the enzyme before DXP [45]. This mechanism is indicative of an underlying conformation change accompanying the binding of NADPH, thereby resulting in the formation of the DXP binding site. Accordingly, relative to DXP, fosmidomycin and FR900098 are competitive inhibitors of MEP synthase, while they are uncompetitive with respect to the binding of NADPH [45] [33] (Figure S5). Hence, NADPH must first bind to the enzyme before fosmidomycin/FR900098 can compete with DXP for its binding site.

**Figure 11:**
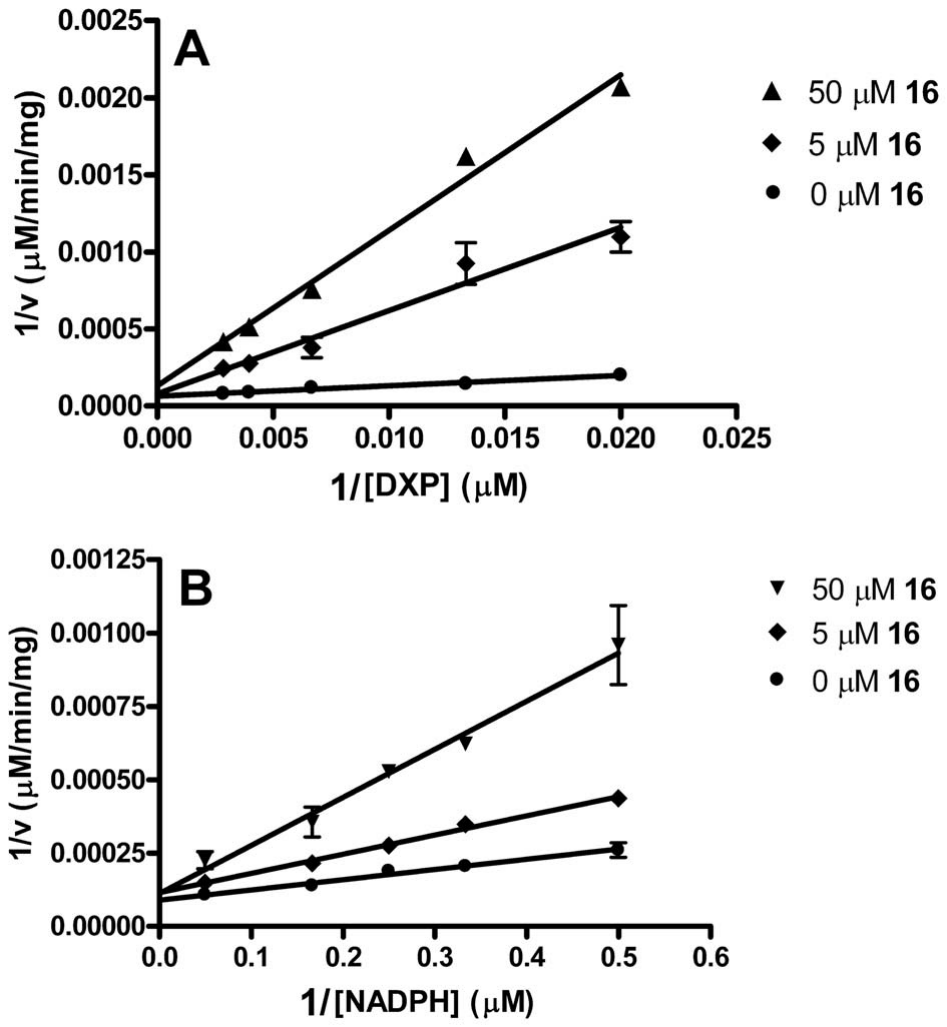
Mode of inhibition by compound 16. The Lineweaver–Burk plots indicate that compound **16** is competitive with respect to DXP (A) and competitive with respect to NADPH (B). All assays were performed in duplicate using purified *Y. pestis* MEP synthase. The enzyme was not preincubated with compound **16**, in contrast to Figure 12.

**Figure 12:**
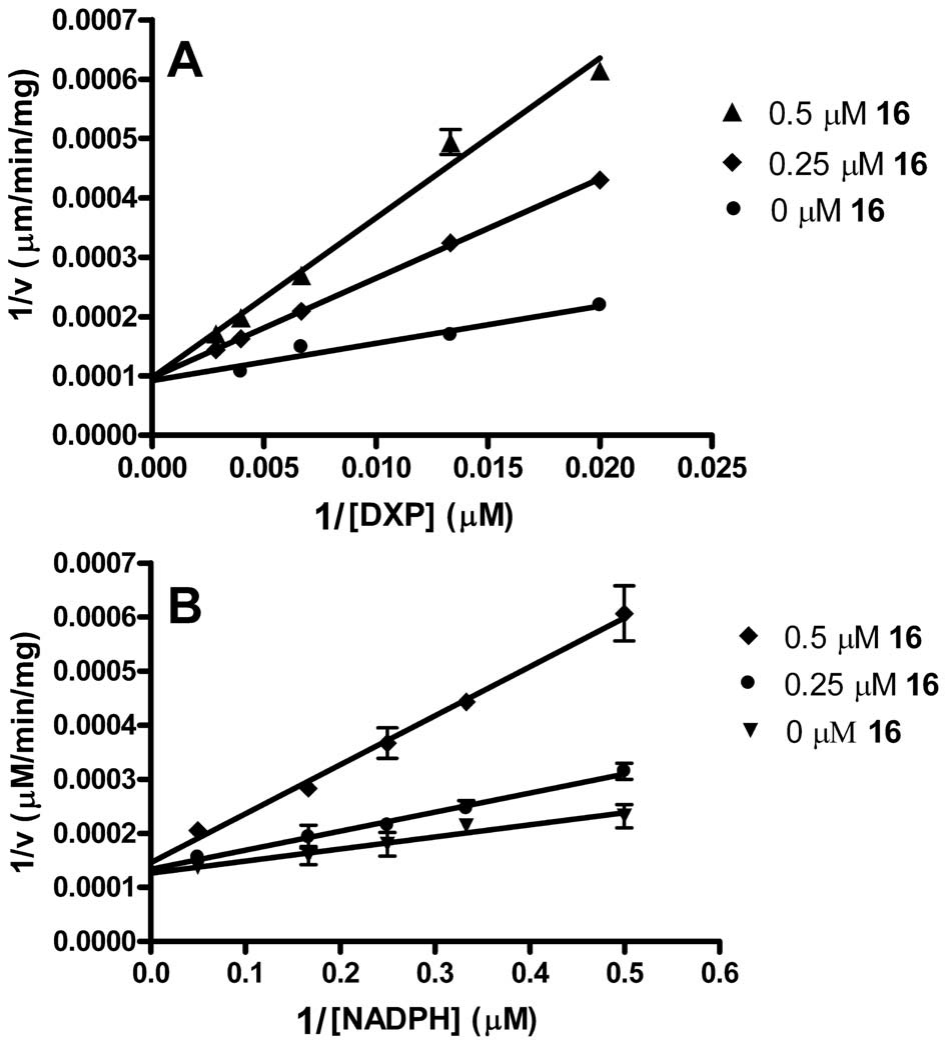
Mode of inhibition with preincubation. When the *Y. pestis* MEP synthase is preincubated with compound **16** (37°C, 10 min) prior to the addition of NADPH and DXP, the Lineweaver–Burk plots still indicate that compound **16** is competitive with respect to DXP (A) and NADPH (B). All assays were performed in duplicate.

In light of the fosmidomycin and FR900098 mechanism of inhibition, and given the anticipated mechanism for the bisubstrate inhibitor **16**, we performed mode of inhibition assays in each of two ways; the first with **16** added after preincubating the enzyme with NADPH (Figure 11) and the second with compound **16** preincubated with the enzyme prior to the addition of any other substrates (Figure 12). As illustrated in Figures 11 and 12, compound **16** is competitive with respect to DXP and competitive with respect to NADPH, under either of the two assay conditions. Thus, in contrast to fosmidomycin and FR900098, compound **16** does not require the initial binding of NADPH to the enzyme. In fact, as it competes with NADPH for a binding site, its activity is more potent when preincubated with MEP synthase in the absence of NADPH (contrast the concentrations of **16** used in the plots shown in Figures 11 and 12). Due to its ability to bind to the NADPH site, compound **16** appears capable of promoting the same structural change in the enzyme as does NADPH, causing the ensuing formation of the DXP binding site. Consequently, compound **16** behaves as a tightly bound inhibitor, binding to the NADPH site and causing a conformation change that subsequently “locks” the inhibitor into the DXP site. Further exploration of this mechanism is currently underway.

**Figure 13:**
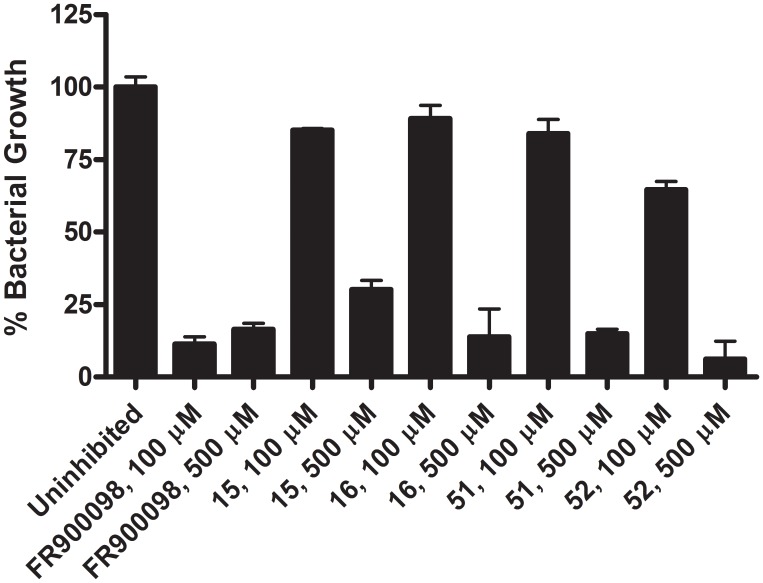
Growth inhibition assay with liquid cultures of *Y. pestis*. *Y. pestis* A1122 was cultured in the presence of either 100 µM or 500 µM of the indicated inhibitor. Bacterial growth is relative to an uninhibited culture. All assays were performed in triplicate. At 500 µM, compounds **15**, **16**, **51**, and **52** have inhibitory activity comparable to FR900098, however relatively poor inhibitory activity is observed at 100 µM. See text for further discussion.

Satisfied that the *Y. pestis* MEP synthase performed well in the pilot scale high throughput screen, we next sought to complete our evaluation of the rational library by determining if compounds **15** and **16** would make good lead molecules for subsequent drug development. Secondary to the enzyme assays, compounds **15** and **16** were next evaluated in a growth inhibition assay with liquid cultures of *Y. pestis* A1122. As shown in Figure 13, relative to FR900098, compounds **15** and **16** demonstrate measurable but modest inhibitory activity towards *Y. pestis*. Given the potent activity of these compounds towards the isolated enzyme, we speculated that the relatively poor activity towards whole cells might reflect cell permeability limitations. However, as seen in Fig. 13, inhibitory activity is not dramatically improved with lipophilic ester derivatives of compound **15** (compounds **51** and **52**), suggesting that compound efflux and/or intracellular instability may impact the effective concentration of the inhibitor. While further investigation is required to deduce the specific details pertaining to the activity of compounds **15** and **16**, our pilot scale screening of a rational library demonstrates the amenability of the *Y. pestis* MEP synthase to high throughput screening (and underscores the importance of the secondary screen in lead molecule selection).

### Natural Product Library Screening

**Figure 14:**
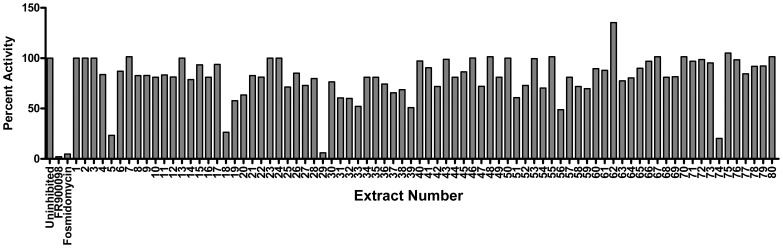
Screening of a natural product library. The *Y. pestis* MEP synthase was assayed in the presence of ca. 75 µg/mL of the indicated extract. Residual activity is relative to the assay performed with vehicle alone (DMSO). Assays with fosmidomycin and FR900098 were performed with 100 µM inhibitor. All assays were performed in duplicate. Of those compounds tested, four inhibit the enzymatic activity by >75%, including e5, e18, e29, and e74. The activity of e29 is comparable to that of fosmidomycin.

**Figure 15:**
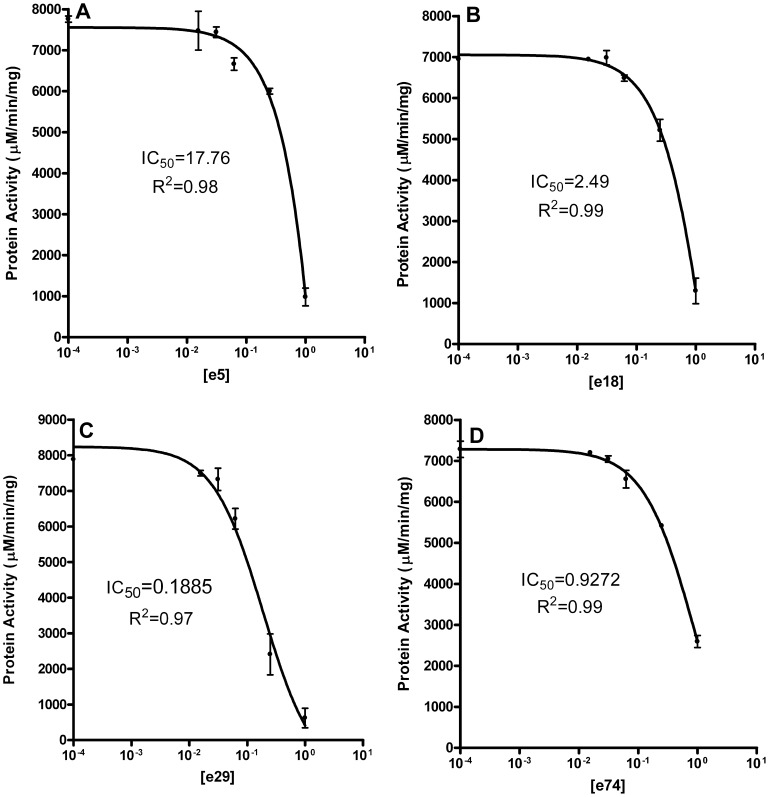
Dose-response plots of the *Y. pestis* MEP synthase with the top four natural product extracts. The enzyme was assayed with various concentrations of A) e5, B) e18, C) e29, or D) e74 and the resulting activity plotted as a function of extract concentration. The concentration of each extract stock solution, ca. 15 mg/mL, is defined as 1 and serial dilutions from these stocks were then used for the analysis. R^2^ and IC_50_ values are indicated. All assays were performed in duplicate.

**Figure 16:**
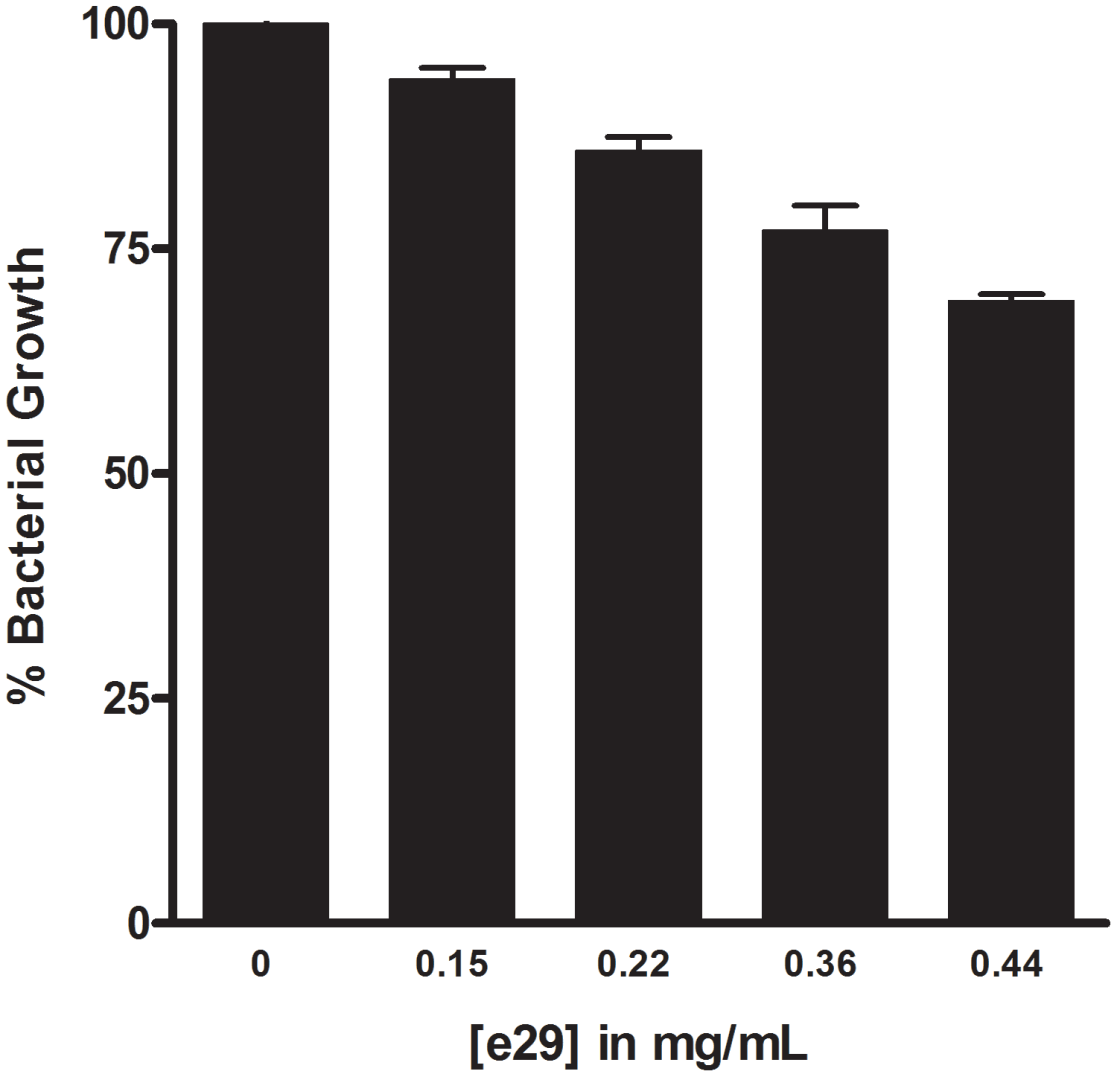
*Yersinia pestis* growth inhibition assay with extract 29. *Y. pestis* A1122 was cultured in the presence of e29 at the indicated concentrations. Bacterial growth is relative to an uninhibited culture. All assays were performed in duplicate. The extract inhibits bacterial growth in a dose-dependent fashion.

In addition to screening a rationally designed molecular library, we also screened our proprietary natural product library for inhibitory activity against the purified *Y. pestis* MEP synthase. This library contains 80 different extracts obtained from widely diverse biological sources. Each individual extract consists of a complex mixture of metabolites (a metabolome) isolated via a non-targeted organic extraction. As shown in Figure 14, four of the 80 extracts demonstrate significant inhibitory activity (>75% inhibition). Subsequent dose-response assays confirm this inhibitory activity and identify extract 29 (e29) as the most potent inhibitor among the four (Figure 15). A follow-on *Y. pestis* growth inhibition assay demonstrates the dose-dependent activity of e29 (Figure 16).

**Figure 17:**
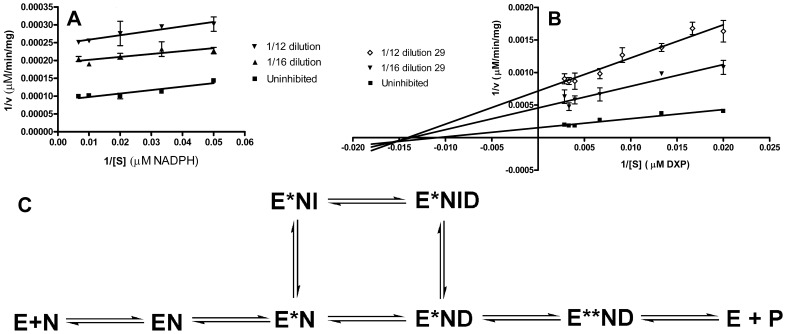
Mechanism of inhibition by e29. A) Relative to NADPH, e29 is an uncompetitive inhibitor of the purified *Y. pestis* MEP synthase. B) Relative to DXP, e29 is a noncompetitive inhibitor. C) A model of e29 inhibition. MEP synthase (E) undergoes a conformational change (E*) upon binding of NADPH (N), exposing an allosteric site to which the inhibitor (I) binds. As the inhibitor is noncompetitive with respect to DXP (D), I may bind the E*N or E*ND complex, thereby inhibiting the enzyme.

In light of its inhibitory activity, we next determined the e29 mechanism of action using the purified *Y. pestis* MEP synthase (Figure 17). While FR900098 is uncompetitive with respect to NADPH and competitive with respect to DXP (Figure S5), the binding of e29 is uncompetitive with respect to NADPH but noncompetitive with respect to DXP. As this represents an entirely new class of MEP synthase inhibitor, we performed additional, confirmatory assays with purified recombinant *F. tularensis* and *M. tuberculosis* MEP synthase, the results of which corroborate this discovery (Figure S6). Hence, e29 contains an active compound that serves as an allosteric inhibitor of MEP synthase (Figure 17C). To date, no such inhibitors of MEP synthase have been reported, making the inhibitory compound in e29 a novel class of inhibitor, and suggesting a new binding site on MEP synthase amenable to rational drug design. As MEP synthase regulates metabolic flux through the MEP pathway [53], we speculated that isopentenyl pyrophosphate (IPP), dimethylallyl pyrophosphate (DMAPP), and/or geranyl pyrophosphate (GPP) might serve as feedback inhibitors of the enzyme. Hence, we assayed the *Y. pestis* MEP synthase in the presence of each of these individual compounds at concentrations of 100, 500, and 1000 µM. No enzyme inhibition was observed under any of the assay conditions (data not shown). Thus, the active component of e29 is not likely to be any of these three isoprenoids. Effort is currently underway to isolate and identify the active component in e29.

## Concluding Remarks

In response to the urgent need for novel antibiotics, MEP synthase has received considerable attention as a target for drug development. The *Y. pestis* MEP synthase demonstrates significant sequence conservation with its orthologous counterparts, therefore leading to a predicted tertiary structure that is comparable to crystallographic-defined structures of MEP synthase. However, structural comparison identifies subtle differences among the MEP synthase family, particularly within the active site of the *M. tuberculosis* homolog, thereby rationalizing the observed differences in measured inhibitor activity. These structural differences warrant further consideration when developing rationally designed broad spectrum antibiotics.

The screening of a rationally designed, synthetic, bisubstrate molecular library, developed from crystal structures of the *M. tuberculosis* MEP synthase, demonstrated the amenability of the *Y. pestis* MEP synthase to a screening campaign and identified several effective inhibitors of the purified enzyme. Subsequent mechanistic assays reveal that the most effective inhibitor (compound **16**) binds to both the NADPH and DXP sites, acting as a potent tight binding inhibitor of the enzyme. However, a growth inhibition secondary screen reveals that the whole-cell inhibitory activity of compound **16** is relatively poor, indicating the need for additional structure−activity relationship studies to elucidate the underlying etiology.

By screening a library of natural product extracts, we identified four biological mélanges containing an inhibitor of MEP synthase. The most potent extract, e29, reduced MEP synthase enzymatic activity by roughly 90% and demonstrated a dose-dependent inhibition of *Y. pestis* growth *in vitro*. While all previously reported MEP synthase inhibitors bind in the active site of the enzyme, the active component in e29 is the founding member of a new class of desirable allosteric inhibitors of the enzyme. Unlike competitive inhibitors of an enzyme, which lose potency as the substrate pools accumulate, an allosteric inhibitor maintains effectiveness while metabolic flux through the pathway is impeded.

## Supporting Information

Figure S1 **Dose-response plot of** ***Y. pestis*** **growth as a function of ampicillin concentration.** Fractional growth is calculated as the ratio of cell density (OD_600_) in the presence of inhibitor to cell density in the absence of inhibitor. Ampicillin is an FDA approved inhibitor of bacterial transpeptidase, resulting in the disruption of cell wall biosynthesis. Nonlinear regression fitting was performed, resulting in an IC_50_ of 10.8 µM (3.8 µg/mL). The goodness-of-fit (R^2^) value is indicated.
(TIF)

Figure S2 Sequence alignment of various MEP synthase homologs using Clustal Omega, where identical residues are denoted by an asterisk (*) and chemically similar residues are denoted by a colon (:). Each residue involved in catalysis [43] is colored based on the substrate or cofactor with which it primarily interacts, with residues in pink associating with NADPH, residues in blue associating with DXP, and residues in yellow coordinating the divalent cation. The serine residue boxed in red was identified in [26] as a possible phosphorylation site used for regulation of the enzyme.
(TIF)

Figure S3 **Structural features of the** ***Y. pestis*** **MEP synthase.** A) Predicted structure of the *Y. pestis* MEP synthase, homology modeled using templates selected by I-TASSER’s threading alignment algorithm. A cartoon representation of the tertiary structure is shown, with alpha helices colored pink, beta sheets colored yellow, and coiled regions colored white. Residues comprising the substrate binding site (colored dark blue with backbone and sidechain residues shown) were identified via primary sequence alignment and the resolved structure of *M. tuberculosis* MEP synthase [43]. B) Overlay of the predicted *Y. pestis* MEP synthase (shown as a cartoon representation) and the resolved crystal structure of the *E. coli* MEP synthase (PBD 2EGH; shown as a purple ribbon). The two structures are highly similar, with a TM-score of 0.996 and a RMSD of 0.46. C) ProQ2 was used to evaluate the quality of the *Y. pestis* MEP synthase model, providing scores ranging from 0 (unreliable) to 1 (reliable). Regions of the model scoring <0.5 are colored light blue in the structure shown in A), and are comprised of residues 1, 301, 303, 397, and 398.
(TIFF)

Figure S4 **Graphical determination of the inhibition constant.** Because fosmidomycin and FR900098 are slow, tight binding inhibitors, the *Y. pestis* MEP synthase was preincubated with the inhibitor for 10 minutes prior to addition of substrate. The absolute value of the X intercept of the line produced from linear regression fitting the plot of K_M_^app,DXP^ as a function of inhibitor concentration defined the K_i_ as 968 nM and 170 nM for fosmidomycin and FR900098, respectively. The R^2^ values are indicated.
(TIF)

Figure S5 **Mode of inhibition by FR900098.** The Lineweaver–Burk plots indicate that FR900098 is uncompetitive with respect to NADPH (A), but competitive with respect to DXP (B). All assays were performed in duplicate using purified *Y. pestis* MEP synthase.
(TIF)

Figure S6 **Mode of inhibition by e29.** The Lineweaver–Burk plots generated from assays with purified *F. tularensis* MEP synthase (A and B) or purified *M. tuberculosis* MEP synthase (C and D) indicate that e29 is uncompetitive with respect to NADPH and noncompetitive with respect to DXP.
(TIFF)
