# Supplementary figures and images for: Kinetic Characterization and Allosteric Inhibition of the Yersinia pestis 1-Deoxy-D-Xylulose 5-Phosphate Reductoisomerase (MEP Synthase)
Source: PLoS One. 2014 Aug 29;9(8):e106243. doi: 10.1371/journal.pone.0106243 (PMC4149570; doi:10.1371/journal.pone.0106243)

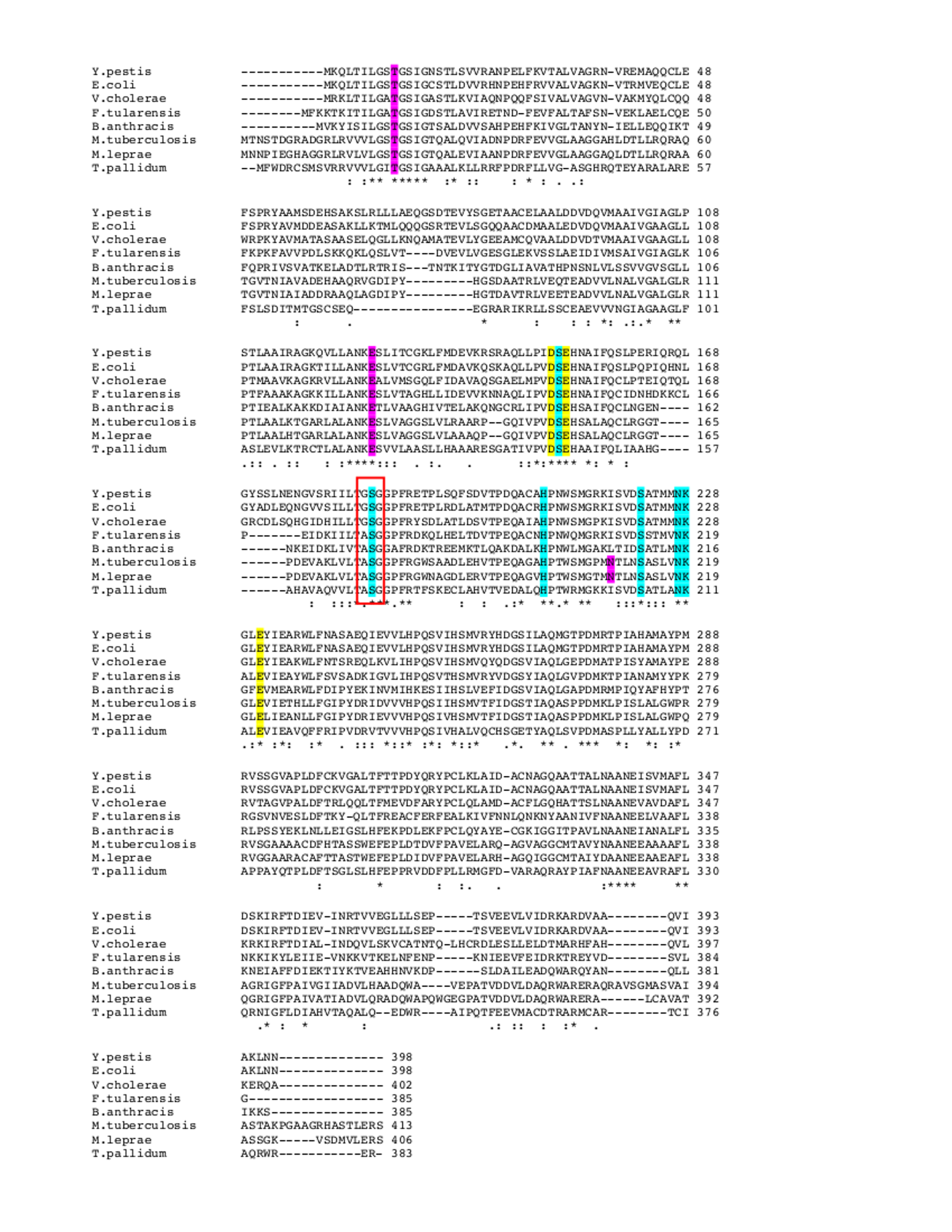

Supplement: Figure S2 — Sequence alignment of various MEP synthase homologs using Clustal Omega, where identical residues are denoted by an asterisk (*) and chemically similar residues are denoted by a colon (:). Each residue involved in catalysis [43] is colored based on the substrate or cofactor with which it primarily interacts, with residues in pink associating with NADPH, residues in blue associating with DXP, and residues in yellow coordinating the divalent cation. The serine residue boxed in red was identified in [26] as a possible phosphorylation site used for regulation of the enzyme. (TIF) [file pone.0106243.s002.tif]

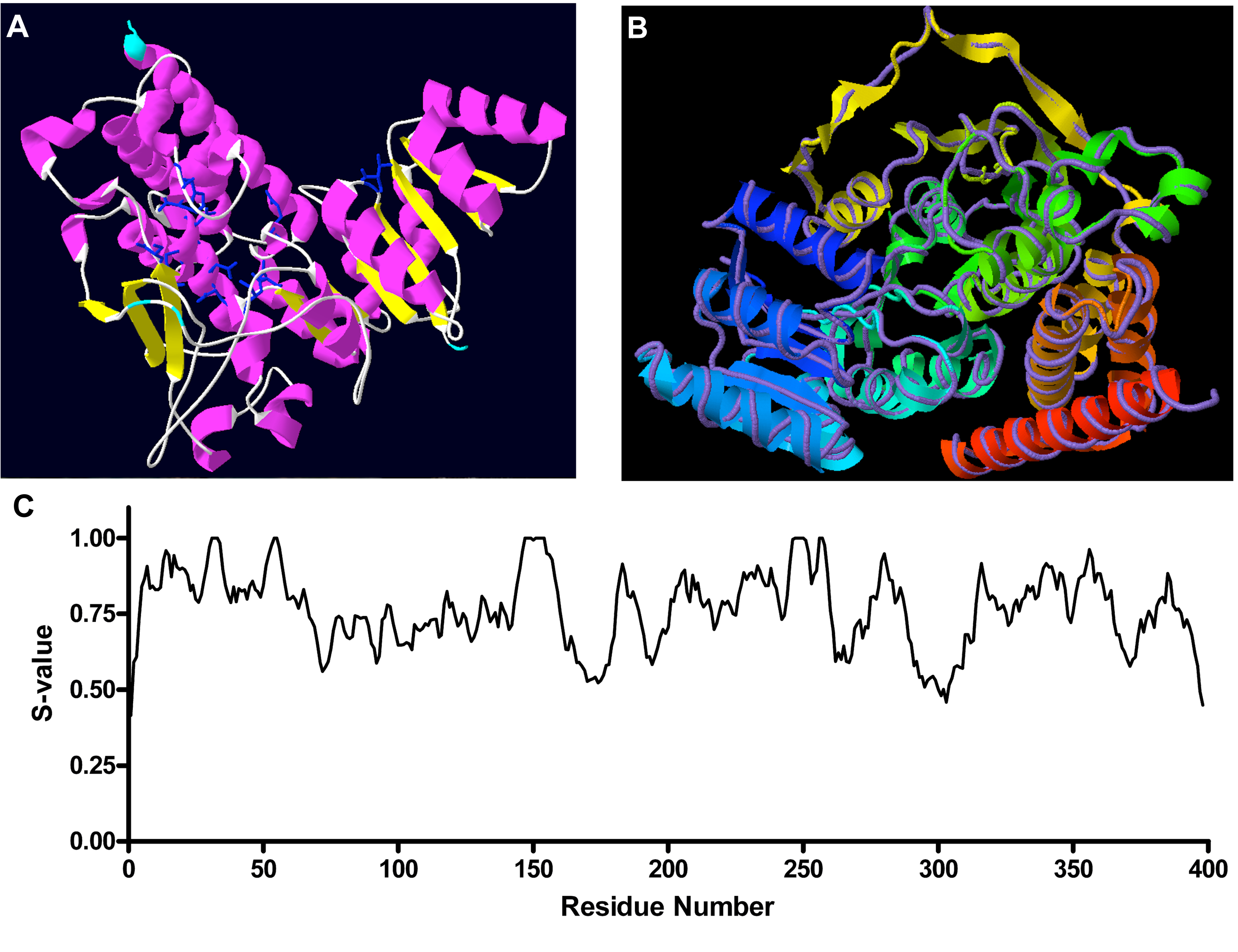

Supplement: Figure S3 — Structural features of the Y. pestis MEP synthase. A) Predicted structure of the Y. pestis MEP synthase, homology modeled using templates selected by I-TASSER’s threading alignment algorithm. A cartoon representation of the tertiary structure is shown, with alpha helices colored pink, beta sheets colored yellow, and coiled regions colored white. Residues comprising the substrate binding site (colored dark blue with backbone and sidechain residues shown) were identified via primary sequence alignment and the resolved structure of M. tuberculosis MEP synthase [43]. B) Overlay of the predicted Y. pestis MEP synthase (shown as a cartoon representation) and the resolved crystal structure of the E. coli MEP synthase (PBD 2EGH; shown as a purple ribbon). The two structures are highly similar, with a TM-score of 0.996 and a RMSD of 0.46. C) ProQ2 was used to evaluate the quality of the Y. pestis MEP synthase model, providing scores ranging from 0 (unreliable) to 1 (reliable). Regions of the model scoring <0.5 are colored light blue in the structure shown in A), and are comprised of residues 1, 301, 303, 397, and 398. (TIFF) [file pone.0106243.s003.tiff]

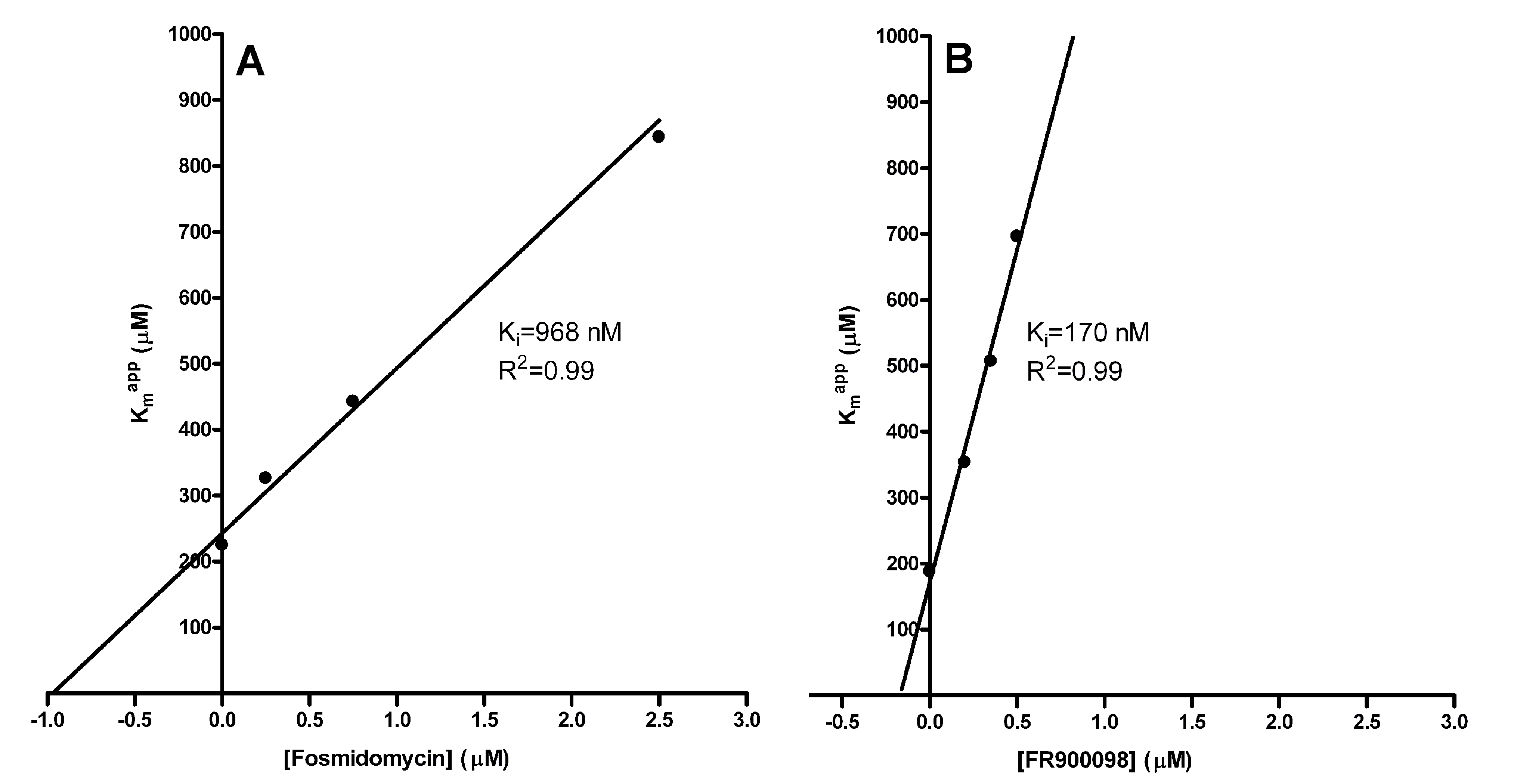

Supplement: Figure S4 — Graphical determination of the inhibition constant. Because fosmidomycin and FR900098 are slow, tight binding inhibitors, the Y. pestis MEP synthase was preincubated with the inhibitor for 10 minutes prior to addition of substrate. The absolute value of the X intercept of the line produced from linear regression fitting the plot of KM app,DXP as a function of inhibitor concentration defined the Ki as 968 nM and 170 nM for fosmidomycin and FR900098, respectively. The R2 values are indicated. (TIF) [file pone.0106243.s004.tif]

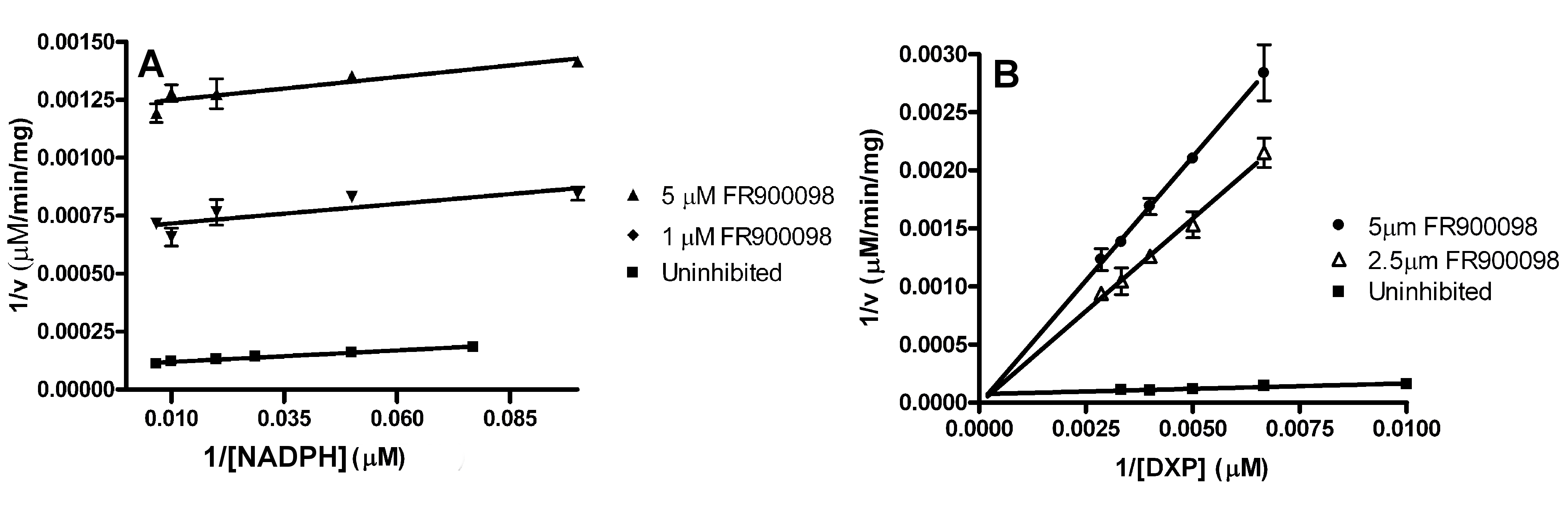

Supplement: Figure S5 — Mode of inhibition by FR900098. The Lineweaver–Burk plots indicate that FR900098 is uncompetitive with respect to NADPH (A), but competitive with respect to DXP (B). All assays were performed in duplicate using purified Y. pestis MEP synthase. (TIF) [file pone.0106243.s005.tif]

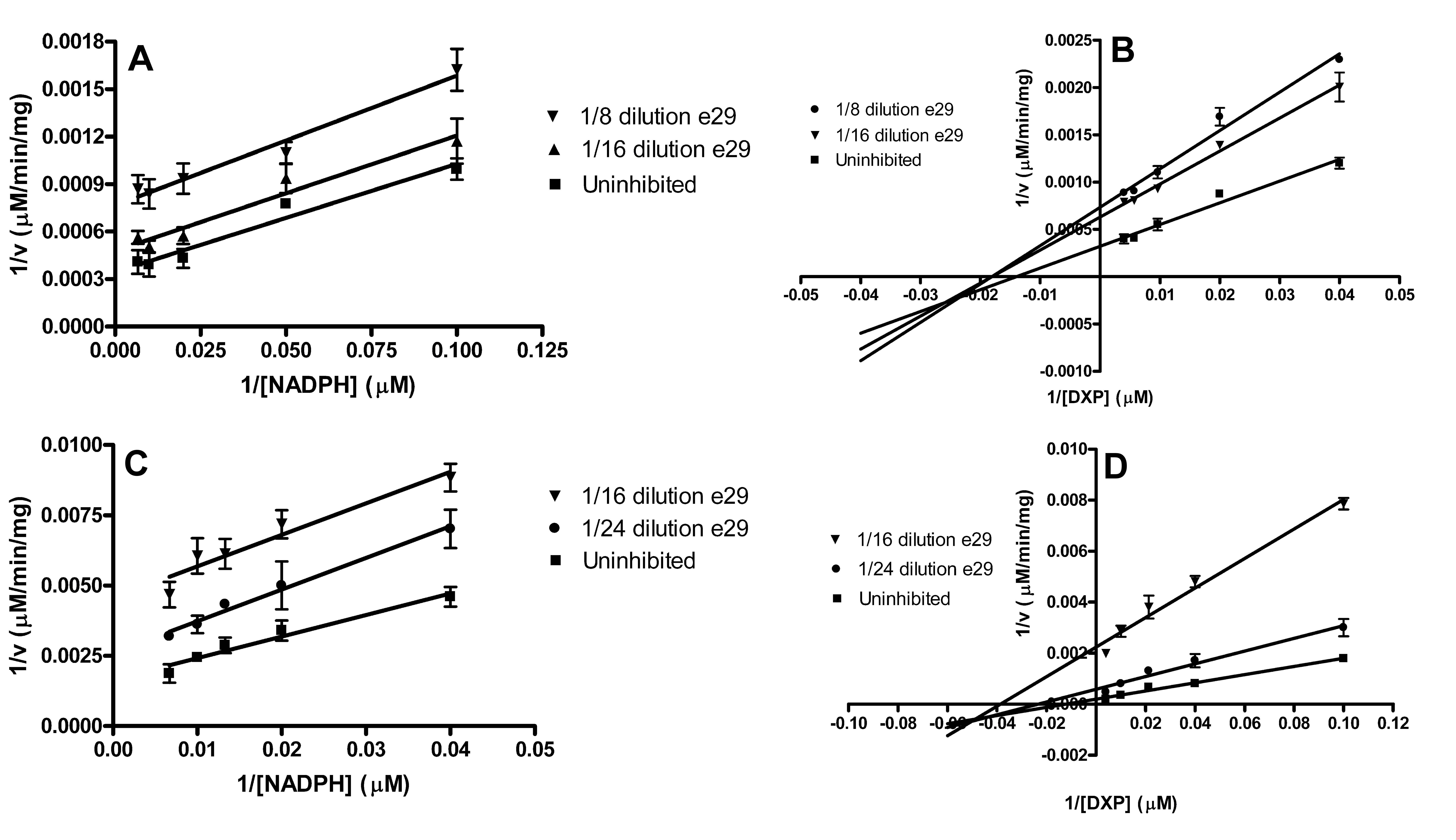

Supplement: Figure S6 — Mode of inhibition by e29. The Lineweaver–Burk plots generated from assays with purified F. tularensis MEP synthase (A and B) or purified M. tuberculosis MEP synthase (C and D) indicate that e29 is uncompetitive with respect to NADPH and noncompetitive with respect to DXP. (TIFF) [file pone.0106243.s006.tiff]
